# A Literature Review of Cutibacterium Acnes: From Skin Commensal to Pathogen in Shoulder Surgery

**DOI:** 10.7759/cureus.69460

**Published:** 2024-09-15

**Authors:** Arshad Iqbal, Muhammad Ali Javaid, Muhammad Sohail, Faiz Khan

**Affiliations:** 1 Trauma and Orthopaedics, Prince Charles Hospital, Cwm Taf Morgannwg University Health Board, Merthyr Tydfil, GBR

**Keywords:** cutibacterium acnes, opportunistic bacterial infection, opportunistic organisms, propionibacterium acnes/ cutibacterium acnes, shoulder arthroplasty, shoulder arthroplasty/ replacement, shoulder surgery, skin commensal

## Abstract

*Cutibacterium acnes*, previously known as *Propionibacterium acnes*, is a gram-positive rod in the pilosebaceous glands and commonly implicated in acne vulgaris. Its role in prosthetic joint infections, particularly in shoulder surgeries, has recently gained attention due to its prevalence around the shoulder girdle. This review collates evidence on the pathogenic role of *C. acnes* in shoulder surgeries, discussing preventive measures, risk factors, clinical presentation, investigation, and treatment strategies. *C. acnes* infections are complex, often presenting with non-specific symptoms and delayed diagnoses. Risk factors include male sex, presence of hair, shoulder steroid injections, and previous shoulder surgeries. Investigations such as inflammatory markers, synovial fluid analysis, diagnostic arthroscopy, tissue cultures, and advanced molecular techniques like next-generation sequencing and multiplex polymerase chain reaction are explored for their effectiveness in detecting *C. acnes*. Treatment strategies range from prolonged antibiotics and antibiotic spacers to single-stage and two-stage revision surgeries. Studies indicate that single-stage revision may provide better outcomes compared to two-stage revision. Effective management of *C. acnes* infections requires careful assessment, relevant investigations, and tailored treatment approaches. This review emphasizes the need for further research to address intraoperative contamination and to develop more efficient diagnostic and treatment methods.

## Introduction and background

*Cutibacterium acnes* (previously *Propionibacterium acnes*) is a gram-positive rod and a known skin commensal. This organism resides in the pilosebaceous gland and is commonly implicated in the skin condition of acne vulgaris. *C. acnes* has gained recent attention due to its presence in prosthetic joints, particularly in shoulder surgeries and replacements. This increased attention is likely due to the abundance of this organism around the shoulder girdle. In this area, it is relatively unaffected by skin preparation immediately before the skin incision, allowing it to be carried into deeper tissue layers. Indeed, it is so commonly found in the deep tissues around the shoulder in cases without suspected infection that distinguishing between contaminant and pathogen can be extremely difficult [1].

Multiple studies have investigated the risk factors associated with *C. acnes*, commonly reporting male sex [2-11], presence of hair [5,12], shoulder steroid injections [4,13], and previous shoulder surgery [14,15]. Its presentation is often non-specific and can occur long after shoulder arthroplasty, leading to delayed diagnosis [2]. Pain and stiffness are the most reported symptoms [16-18]. Long-term follow-up of *C. acnes* cases has shown osteolysis around shoulder implants [19].

The presence of *C. acnes* in shoulder specimens is complex to understand for several reasons. First, it is challenging to determine whether this organism is a contaminant or actually pathogenic in most cases due to the significant number of positive culture specimens with no specific infective symptoms. Second, blood tests, including white blood cell count, C-reactive protein (CRP), and erythrocyte sedimentation rate (ESR), have low sensitivity for assessing the inflammatory response [2,13,20-22]. Third, *C. acnes* is difficult to culture, and the turnaround time for a positive culture can be up to two weeks or more [1,2,23].

Despite increasing understanding of the pathogenic role of *C. acnes* and techniques to avoid infection, infections still occur, and treatment strategies are evolving as our knowledge expands. This review aims to collate current evidence and provide an update on the understanding of the pathogenic role of *C. acnes* in shoulder surgeries. We will cover preventive measures, risk factors, clinical presentation, investigation, and treatment strategies, offering a comprehensive update on the role of *C. acnes* in shoulder surgeries.

## Review

Search strategy

We conducted a detailed and broad search through the Medline and Embase databases using the key search terms shown in Table 1. We did not apply search limits for language, age, or year of publication. After deduplication, the search yielded a total of 185 studies and reviews, which were further reduced to 157 after additional deduplication. An additional relevant paper was found through reference scanning and added to the total, resulting in 158 studies. We did not apply the population, intervention, comparison, and outcome search strategy due to the broader and basic science nature of this review, as no interventions or outcomes were assessed. This search was carried out on May 15, 2023.

**Table 1:** Search terms.

Step	Search Term
1	Embase 1974 to present
2	Medline (Ovid MEDLINE® Epub Ahead of Print, In-Process & Other Non-Indexed Citations, Ovid MEDLINE® Daily and Ovid MEDLINE®) 1946 to present
3	Propionibacterium acnes or Cutibacterium acnes.mp.
4	Shoulder Joint/ or Arthroplasty, Replacement, Shoulder/ or shoulder replacement.mp.
5	3 and 4
6	Remove duplicates from Step 5

Discussion

*C. acnes* is an anaerobic, gram-positive bacillus, a normal skin commensal, relatively abundant around the shoulder girdle. This bacterium primarily colonizes the pilosebaceous glands of human skin and is a common pathogen identified in postoperative shoulder infections [1,2,6]. Risk factors include young male patients [4,5,9], previous shoulder surgery [14,15], and shoulder injections [13]. Compared to other prosthetic joint infections, *C. acnes* infections are more complex due to their low virulence. These infections may be insidious and manifest years after the initial surgery, resulting in joint arthropathy or prosthesis infection [24]. Clinical findings are subtle [23], usually presenting as persistent shoulder pain or stiffness [16,17,25,26]. Fistula formation has also been reported [27].

Contamination Theory

Current decolonizing techniques have proven ineffective due to *C. acnes*' unique niche within dermal sebaceous glands and hair follicles [28]. The organism likely spreads throughout the surgical field from the subdermal layer via soft tissue handling by the surgeon and instruments [6]. This also explains why routine skin preparation is ineffective in shoulder surgeries. Qui et al. [29] supported the contamination theory, while Patzer et al. [30] refuted it but could not clarify whether *C. acnes* is a commensal or enters the joint through hematological, lymphatic, or unknown pathways.

Polyclonality and Phylotyping

Hsu et al. showed significant differences in the clonality of *C. acnes* subtypes in patients undergoing revision arthroplasty compared to primary arthroplasty, with the SLST subtype-A being the most common in strongly positive revision arthroplasty patients [31]. Similarly, Bumgarner et al. demonstrated the polyclonality of deep tissue samples and suggested that sequence-based characterization of virulence and antibiotic resistance may require testing multiple deep specimens [32].

Another interesting aspect is the hemolytic strains of *C. acnes* and whether they have increased pathogenicity compared to non-hemolytic strains. Mahylis et al. conducted a retrospective analysis of 39 patients who underwent revision surgery and had at least one positive culture [33]. The study concluded hemolysis was not associated with increased pathogenicity in patients with *C. acnes*-positive cultures following revision shoulder arthroplasty. Corvec et al. argued that considering only one positive culture growth is insufficient to identify true infection compared with colonization or contamination and suggested that hemolysis is a phylogenetic rather than a pathogenic marker [34]. Boyle et al. reported that hemolysis might serve as a marker assisting in the diagnosis of infection. They acknowledged Mahylis' findings but commented that the discrepancy could be due to variations in culturing techniques, definitions of infection, and methodologies for observing hemolysis [35]. Additionally, this study showed distinct genetic differences in *C. acnes* strains during infection versus contamination.

Preventive Strategies

Multiple strategies have evolved over the last few decades to reduce bacterial load and avoid seeding into the surgical wound, joint, or prosthetic joint intraoperatively. These strategies can be categorized into several groups for convenience.

Barrier prevention: Barrier prevention aims to isolate the skin to avoid deeper seeding into the wound. Yamakado et al. conducted a randomized controlled trial (RCT) that found significant evidence of lower rates of *C. acnes* cultured during arthroscopic rotator cuff repair using an adhesive drape and chlorhexidine skin preparation [36]. In addition to skin preparation and adhesive drape, Smith et al. studied a wound edge protector drape, significantly decreasing the incidence of *C. acnes* in the surgical field [37]. This, in turn, could reduce the transmission to deeper tissues and lower the *C. acnes* deep infection rate. Lorenzetti et al. added a cyanoacrylate microbial sealant to their revision shoulder surgery patients and reported that this might reduce the prevalence of positive cultures and infection, although further research with greater power is needed to confirm these findings [38].

Skin preparation: Multiple approaches have been taken for skin preparation since *C. acnes* resides in the pilosebaceous glands. Studies have identified axillary hair as a risk factor for *C. acnes* colonization [5,12]. However, removing axillary hair does not affect the burden of *C. acnes* in the axilla [39]. Whether hair removal reduces the chances of *C. acnes* infection is still unproven. Viable *C. acnes* persists within the skin dermis despite standard antimicrobial precautions [40]. One study showed that blue light and a photosensitizer significantly reduce the bacterial burden of periprosthetic joint infection (PJI) shoulder isolates of *C. acnes* [41]. Although commonly used in dermatology, this technique has not been tested in vivo in orthopedic surgeries to show reductions in positive cultures and/or infection rates.

Some surgeons have tried preoperative chlorhexidine showers and benzyl peroxide soaps, but recent studies showed these to be ineffective in reducing *C. acnes* bacterial load [31,42]. Koh et al. quantitatively assessed various methods, including chlorhexidine shower, chlorhexidine scrub, ChloraPrep skin preparations, Iodophor-impregnated adhesive drapes, intraoperative washout with 2 L of saline plus cefazolin and gentamicin, application of betadine around the wound just before closure, and perioperative cefazolin. None of these measures effectively eliminated *C. acnes* colonization [5]. These findings suggest that *C. acnes* cannot be eliminated due to its presence deeper within the dermal layers in the pilosebaceous glands rather than just on the skin surface. The bacteria infiltrate the wound and deeper tissues from the dermal layer, which is difficult to eliminate with surface skin preparations.

This led Sabetta et al. to use benzoyl peroxide (BPO) cream, commonly used for treating acne vulgaris. Their findings were encouraging, showing that BPO effectively reduced *C. acnes* on the skin at the beginning and end of a surgical procedure [43]. Subsequent studies reported better eradication and less colonization with BPO in addition to routine perioperative prophylaxis [44-46]. This could potentially reduce the chances of perioperative infection. An RCT comparing BPO alone versus BPO plus chlorhexidine did not show any superiority of either, but in this trial, BPO was applied just before surgery, unlike the preoperative 48-hour application by Sabetta et al. [47]. Dizay et al. used BPO plus clindamycin gel and reported 66.7% and 78.9% effectiveness in eliminating superficial colonization with one and more than one application, respectively. Similarly, deep colonization was reduced to 3.1% and 0% with one and more than one application, respectively [48].

Recent studies have investigated inexpensive hydrogen peroxide, which has been shown to reduce the number of positive perioperative cultures for *C. acnes*. Although these studies had smaller sample sizes, they provided significant results suggesting that hydrogen peroxide should be considered a reasonable skin preparation for decolonizing *C. acnes* [49,50]. BPO and hydrogen peroxide were subjected to break-even analysis and found suitable for infection prevention for patients undergoing shoulder arthroplasties [51].

Perioperative antibiotics: *C. acnes* is difficult to eradicate and can form biofilms, which enable it to resist antibiotics. A recent systematic review concluded that the optimal perioperative antibiotics for *C. acnes* prophylaxis are controversial [11]. Routine perioperative use of cefazolin or vancomycin has shown lower infection rates than clindamycin used in penicillin-resistant patients [52]. Vancomycin has also shown comparable efficacy in eliminating *Propionibacterium* acnes biofilm as doxycycline and penicillin in an in vitro model [53]. Although doxycycline has been considered, a recent RCT did not show a significant reduction in *C. acnes* colonization with seven days of preoperative doxycycline compared to no preoperative antibiotics [54]. Another RCT added intravenous doxycycline to routine preoperative cefazolin, and again, the addition of doxycycline did not significantly reduce *C. acnes* culture positivity [55]. It appears that doxycycline is effective against *C. acnes* in vitro but not in vivo. Topical benzylpenicillin applied to the wound has a low minimum inhibitory concentration for *C. acnes* and is not significantly chondrotoxic in vitro [56], but further research is needed to assess its safety for in vivo use.

Investigation

Investigating *C. acnes* prosthetic shoulder joint infections is challenging due to non-specific, low-grade symptoms, a lack of inflammatory response similar to other PJIs, and the difficulty of culturing this organism. Additionally, distinguishing positive cultures due to contamination versus infection is problematic [57]. After reviewing patient demographics, history, and examinations, surgeons typically have several options for further investigation.

Inflammatory markers (white blood cell count, erythrocyte sedimentation rate, C-reactive protein): These markers are commonly used in hip and knee joint infections and other orthopedic infections. However, they are less sensitive in the case of *C. acnes* infections in the shoulder [22].

Synovial fluid analysis: In synovial fluid analysis, synovial fluid is collected for various investigations. One such investigation is the calprotectin level. Wouthuyzen et al. showed a 97% negative predictive value in chronic infection, which may prove promising in excluding *C. acnes* infection [58]. Another marker, synovial alpha-defensin (ALDF), has been used to detect PJIs in hips and knees. Recently, Weigelt et al. studied synovial ALDF levels in the context of shoulder joint infection and found the sensitivity, specificity, and positive and negative predictive values to be 60%, 83%, 43%, and 91%, respectively. The overall accuracy was 79%. They concluded that the ALDF test does not appear useful in predicting shoulder PJIs but may help reject these infections [59].

Cytokine analysis is another important aspect of synovial fluid investigation. Cytokines are released by inflammatory cells in response to infections and are elevated in infected tissues and fluids. Frangiamore et al. showed that synovial interleukin (IL)-6, granulocyte-macrophage colony-stimulating factor, interferon-γ, IL-1β, IL-2, IL-8, and IL-10 were significantly elevated in cases of revision shoulder arthroplasty classified as infected. They found that cytokines, both in isolation and combination, were more effective in detecting infection than ESR and CRP in diagnosing prosthetic shoulder infections [60].

Diagnostic arthroscopy: Diagnostic arthroscopy allows a comprehensive assessment of implants, the rotator cuff, native articular surfaces, and scar tissue, as well as obtaining biopsy specimens for indolent infection in patients considering revision arthroplasty surgery [61]. Dilisio et al. reported 100% sensitivity and 100% specificity for diagnosing shoulder infection and identifying the causative organism through arthroscopic shoulder tissue biopsy [62]. However, they based the diagnosis on a single positive culture, which may overestimate infections. Akgun et al. showed that considering at least two positive samples with the same microbiologic growth in arthroscopic biopsies increased specificity and positive predictive value to 94.4% and 80%, respectively, although sensitivity and negative predictive value dropped to 80% and 94.4%, respectively [63]. Thus, shoulder arthroscopy is a valuable tool in investigating prosthetic shoulder infections. Findings from cultures and fresh-frozen sections at revision have been consistent with arthroscopic findings [64].

Tissue culture: Tissue culture has been the most important investigation in identifying *C. acnes* contamination versus infection. This process is challenging for two reasons. First, *C. acnes* is a slow-growing organism, yielding positive cultures in two or more weeks [1,23]. Implementing routine 10-day anaerobic cultures recently improved the recovery rate for *C. acnes* from shoulder specimens [65,66]. Secondly, there is some consensus that a single positive culture is widely considered a contaminant, whereas two positive cultures are probably considered infections [67-69]. Table 2 provides an overview of tissue culture interpretation [67].

**Table 2:** Periprosthetic shoulder infection criteria. ^a^Preoperative finding of infection: preoperative clinical signs (swelling, sinus tract, redness, or drainage), positive erythrocyte sedimentation rate, or positive C-reactive protein. Intraoperative finding of infection: intraoperative gross finding (purulent drainage or necrosis) or positive intraoperative frozen section for acute inflammation.

Category	Criteria^a^
Definite infection	≥1 positive preoperative or intraoperative finding of infection and >1 positive culture, or 1 positive preoperative (aspirate) culture and 1 positive intraoperative culture with the same organism identified [67]
Probable infection	≥1 positive preoperative or intraoperative finding of infection and 1 positive culture, or 0 preoperative or intraoperative findings of infection and >1 positive culture [67]
Probable contaminant	0 preoperative or intraoperative findings of infection and 1 positive culture [67]
No evidence of infection	0 preoperative or intraoperative findings of infection and 0 positive cultures [67]

Thioglycolate broth: Bossard et al. provided detailed information on the sensitivities and specificities of various culture media. Thioglycolate broth stood out with the highest sensitivity rate of 66.3%; however, its specificity of 79.1% was lower than that of direct aerobic and anaerobic media [66]. This study recommended that biopsy specimens from bone and joint infections be cultured to detect *C. acnes* for 10 days with a blind subculture at the end. Shannon et al. reported a faster time to positivity when thioglycolate broth is used anaerobically [66,70].

Frozen sections: Frozen section histology has been used previously to diagnose hip and knee infections. Grosso et al. studied a group of patients with periprosthetic shoulder infections and reported lower sensitivity for *C. acnes* (50%) than infections with other organisms (67%) and 100% specificity using routine histologic grading of frozen sections. Sensitivity increased to 72%, with specificity remaining at 100% when an optimized threshold of 10 polymorphonuclear leukocytes in five high-power fields (400x) was used [71]. An extensive review supported the idea that frozen sections should be considered when diagnosing infection, even in the presence of clinical suspicion [21]. Guild et al. found that arthroscopic frozen section and culture results had a 100% correlation with open frozen section and culture results in patients who had cultures obtained [64].

Implant sonication: In implant sonication, low-intensity ultrasound waves disintegrate biofilm from implants, yielding fluid samples that are subsequently sent to the laboratory for microbial culture. This technique has been investigated for shoulder prosthetic joint infections and showed no significant benefit over standard intraoperative cultures [72]. Akgun et al. reported that tissue cultures provide higher sensitivity and specificity than implant sonication in diagnosing shoulder PJI and should remain the gold standard for microbiological diagnosis of shoulder PJI [73]. Sonication would add to surgical time and costs without providing any added benefits.

Molecular Techniques

Next-generation sequencing: Next-generation sequencing (NGS) can identify bacterial genomes and provide detailed insight into the microbiome and pathogenesis. Namdari et al. studied this technique in shoulder PJI and found poor concordance with culture data [74]. Additionally, they found that culture data from revision shoulder arthroplasty cases commonly yield monomicrobial results, whereas NGS data suggest that bacterial loads in revision arthroplasty are most commonly polymicrobial. Rao et al. reported similar findings, showing that NGS is more likely than culture to identify polymicrobial environments, but the best interpretation of these polymicrobial results is currently unknown [75].

Multiplex polymerase chain reaction: Multiplex polymerase chain reaction (PCR) is actively being researched in PJI but is not widely used. The aim is to assess sensitivity and specificity and identify strains that are contaminants versus pathogens. Multiplex PCR of sonication fluid is more sensitive (96.29% vs. 70.37%) and specific (100% vs. 93.65%) than culture methods [76]. Nguyen et al. used a more robust six-gene multiplex PCR, and their study did not show any phylogenetic association with PJI, nor did they find genetic differences between strains causing PJI and those not causing PJI [77]. Holmes et al. combined the high sensitivity of PCR with the specificity of restriction fragment length polymorphism mapping to identify *C. acnes* in surgical isolates before contaminant colonies can grow. This reproducible assay allows surgeons and pathologists to determine if a biopsy is *C. acnes* positive within 24 hours of sampling [78].

Treatment

Managing *C. acnes* infections requires well-thought-out preventive and definitive medical and surgical interventions. This necessity has led to the development of a spectrum of management possibilities, ranging from conservative management to surgical intervention.

Phipatanakul et al. reported two cases of *C. acnes* prosthetic shoulder infection followed for a minimum of six years [19]. Both patients showed good shoulder function and declined resection arthroplasties or radical surgical interventions. Another case report described chronic non-settling oligoarthritis despite six months of antibiotics and nonsteroidal anti-inflammatory drugs [79]. These cases highlight the slow onset and undulant course of *C. acnes* infection and the poor response to antibiotic therapy. Rouleau et al. reported that antibiotic-calcium sulfate-impregnated beads showed high in vitro efficacy against pathological *C. acnes* strains [80]. Their study also indicated that penicillin and rifampicin had the largest inhibition zone diameters and no cases of resistance, suggesting potential efficacy for non-surgical treatments pending further clinical trials. The Infectious Diseases Society of America suggests that in penicillin-allergic patients, combining vancomycin or clindamycin with rifampin is a viable alternative, with the antimicrobial treatment duration typically paralleling that of penicillin co-administered with rifampin, generally extending from 2 to 6 weeks, depending on the infection's severity and the patient's clinical response [81]. An experimental study using a foreign-body infection model demonstrated that the concurrent use of rifampin and daptomycin was the most active regimen against *C. acnes* biofilms [82].

Prolonged antibiotics and antibiotic spacers provide a high rate of eradication and patient satisfaction but may result in lower functional outcomes [83]. Another study reported reasonable shoulder function and minimal pain with long-term antibiotic cement spacers [84]. This approach could be promising for eradication, leading to safer definitive surgical interventions in the future, or it could serve as definitive management for high-risk surgical patients due to age and comorbidities.

**Figure 1:**
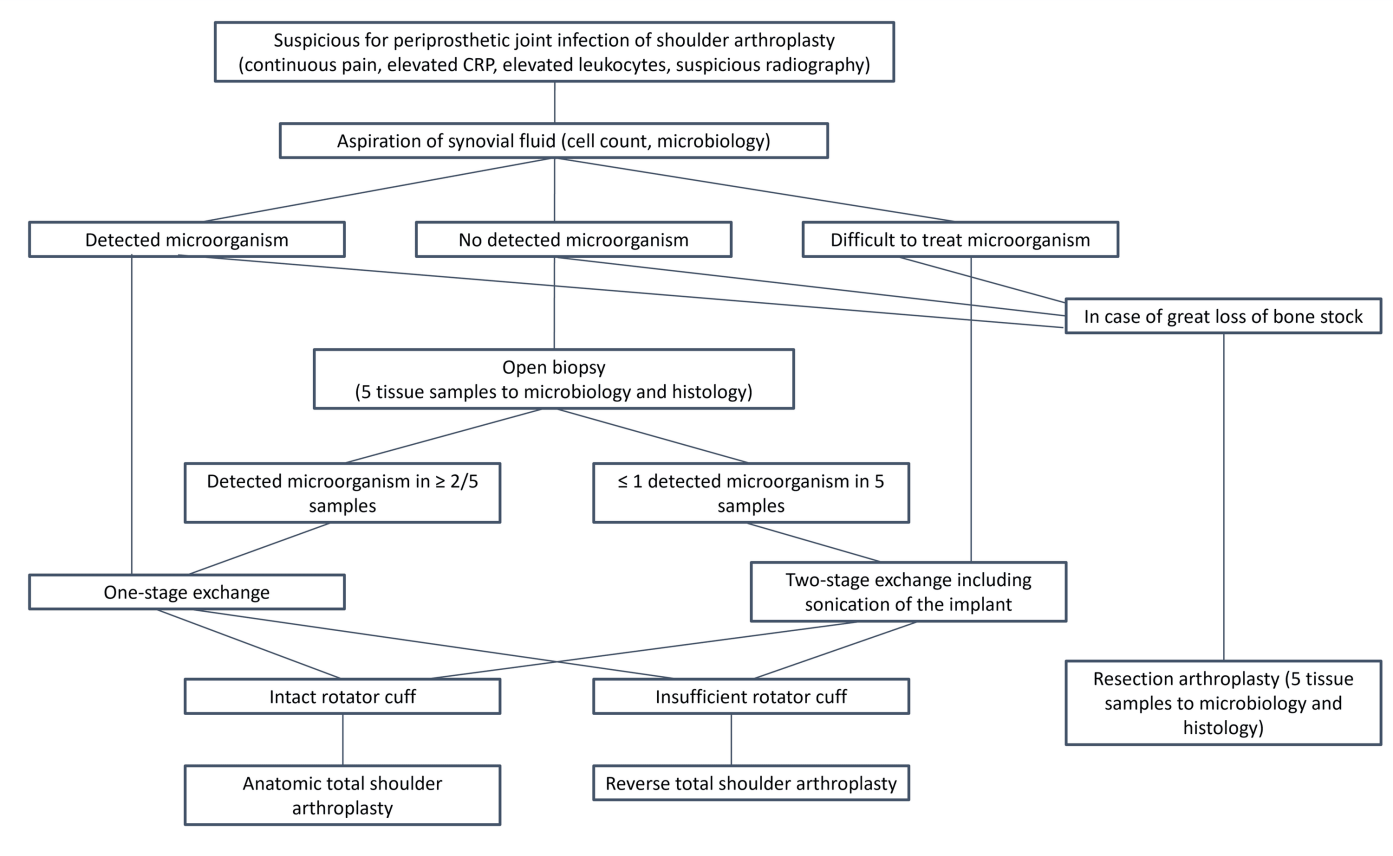
Treatment algorithm for infected shoulder arthroplasty. CRP: C-reactive protein.

Emerging studies suggest that single-stage revision provides good results regarding infection-free survival and function [27,85-87]. Although some studies are retrospective and controlling for confounding factors is challenging, Sevelda et al. conducted a prospective case series with a mean follow-up of 5.8 years and a small sample size of 14 patients [85]. They performed joint tissue sampling and microorganism isolation before surgical intervention and provided targeted antibiotic therapy for six weeks (local, oral, intravenous) with single-stage revision. The infection-free survival rate was 100% at one year and 93% (95% confidence interval) at five years. Figure 1 presents the suggested treatment strategy [85]. Beekman et al. studied a longer course of antibiotics (median duration of three months) and the placement of gentamicin-laden cement intraoperatively without identifying the underlying microorganism before surgery. Despite differences in study design, they reported reliable infection eradication and better functional outcomes with single-stage revision [27].

Similar findings have been reported by Grosso et al. [86] and Junghans et al. [87], whereas Buchalter et al. [88] indicated that two-stage revision could be a viable option for intractable chronic periprosthetic shoulder infections. However, their findings showed a 42% complication rate, with 26% being recurrent infections, higher than the rates reported for single-stage revisions. Recent reviews support single-stage revision for shoulder PJI as an effective treatment for *C. acnes* [89,90].

## Conclusions

This review aimed to summarize current literature relevant to *Cutibacterium acnes *and its role in the pathogenesis of shoulder surgeries. This organism once considered a contaminant, is now considered significantly pathogenic in shoulder surgery, necessitating special perioperative considerations. Identified risk factors include male sex, presence of hair, shoulder injections, and previous surgeries or arthroscopies. Clinical findings usually include chronic pain, stiffness, sinus formation, and prosthetic implant loosening. Investigations include inflammatory markers, synovial fluid and tissue culture, arthroscopic assessment, and advanced molecular techniques such as next-generation sequencing and multiplex PCR. Treatment options range from conservative management with antibiotics to single-stage or two-stage revision surgeries, with or without antibiotic spacer devices, depending on the patient's circumstances.

Effective management requires careful assessment, a low threshold of suspicion for *C. acnes* infection, relevant investigations, and appropriate treatment algorithms. The evolving understanding of *C. acnes* has shifted from viewing it as a skin commensal to recognizing it as a pathogenic organism. Significant progress has been made in identifying risk factors, clinical features, relevant investigations, and treatment options. However, intraoperative contamination from the dermal skin layer remains a challenge, and further research is needed to identify pathogenic strains, understand pathogenic gene expression, and develop investigations with shorter turnaround times to enable timely and specific treatments for better outcomes.
